# The Prone-Transpsoas Approach for Single-Position Lateral Corpectomy: A Case Series

**DOI:** 10.3390/brainsci16060616

**Published:** 2026-06-08

**Authors:** James G. Lyman, Michael C. Oblich, Rishi Jain, James M. Mossner, Najib El Tecle, Kevin Swong

**Affiliations:** 1 Feinberg School of Medicine, Northwestern University, Chicago, IL 60611, USA; james.lymaniii@northwestern.edu (J.G.L.); michael.oblich@northwestern.edu (M.C.O.);; 2 Department of Neurological Surgery, Northwestern University, Chicago, IL 60611, USAkevin.swong@nm.org (K.S.)

**Keywords:** prone-transpsoas approach, single-position, lateral corpectomy, thoracolumbar corpectomy, lumbar fusion, spinal deformity, burst fracture, compression fracture, revision spine surgery, sagittal alignment correction, expandable cage, intraoperative navigation, complex spinal pathology, case series, surgical technique

## Abstract

**Highlights:**

**What are the main findings?**

- In this seven-patient series, prone-transpsoas corpectomy was feasible and safe across trauma, deformity, and revision indications.
- In cases needing deformity correction (*n* = 3), PTP-corpectomy achieved substantial sagittal-plane correction (average Cobb change = 35.1 ± 9.7°).

**What are the implications of the findings?**

- Prone-transpsoas corpectomy allows single-position circumferential access for thoracolumbar corpectomy without patient repositioning.
- All cases were completed without major complications and all maintained postoperative stability with subjective improvements to pain and function at latest follow-up.

**Abstract:**

**Objective:** To describe the surgical technique and early clinical outcomes of prone-transpsoas single-position corpectomy (PTP-corpectomy) for the management of complex thoracolumbar spinal pathology. **Background:** PTP-corpectomy is an emerging technique for providing simultaneous lateral and posterior spinal access without patient repositioning. The previous literature describes the PTP approach for interbody fusions; however, evaluation of its use for corpectomy is limited. This case series reports our experience with the PTP-corpectomy procedure at our institution. **Methods:** We retrospectively reviewed seven patients who underwent PTP-corpectomy surgery for complex spinal pathologies, including severe kyphoscoliosis, traumatic burst fractures, and revision in 2022–2025. Collected variables included demographics, comorbidities, surgical history, perioperative details, radiographic imaging, and clinical outcomes. **Results:** All seven patients successfully underwent PTP-corpectomy. The average operative time was 460.6 ± 147.1 min, and the estimated blood loss (EBL) was 892.9 ± 898.3 mL. Average length of stay (LOS) postoperatively was 6.7 ± 3.0 days. One case required revision of a preexisting construct and complex wound closure with plastic surgery, which had significantly increased operative time and blood loss (767 min, 2700 mL). Excluding this complicated case, the average time was 409 ± 63.7 min, and EBL was 591.7 ± 454.3 mL. All seven patients maintained clinical stability postoperatively, demonstrating improvements in pain and functional status at latest follow-up. Follow-up time ranged from 41 to 375 days. **Conclusions:** Our experience adds to the limited body of evidence that the PTP approach is well suited for corpectomy procedures, and that it is feasible, safe, and effective at improving clinical outcomes for complex spinal pathologies. This series adds to the limited case volume describing this technique in the current literature. Future studies with larger patient populations are warranted to further validate these findings.

## 1. Introduction

The prone-transpsoas (PTP) approach has gained interest as an effective and surgically efficient alternative to dual-position lateral lumbar interbody fusion (LLIF) when circumferential access to the vertebral column is necessary [1,2,3]. The technique was first reported by Pimenta et al. in 2020 and involves simultaneous lateral and posterior access to levels of interest, all with the patient in the prone position [4]. A compelling use of the PTP is single-position corpectomy (PTP-corpectomy). Several groups have discussed corpectomy with a position change [5,6] or solely in the lateral decubitus position [7,8,9]. However, the use of the PTP approach for single-position corpectomies has not been robustly described in the literature.

Since corpectomy in the thoracic and lumbar regions often benefits from circumferential access, PTP-corpectomy is particularly compelling as it provides necessary access without the need for repositioning [2,10]. Many of the benefits of the PTP approach for fusion, described by Pimenta et al. and additional studies, apply to corpectomy as well [1,2,3]. These include advantageous positioning of anterior soft tissue structures, potential for greater sagittal-plane correction, and improved operative efficiency, as surgeons can work in tandem laterally and posteriorly [11,12,13]. For corpectomy in particular, the PTP approach enables a large access corridor as gravity displaces anterior structures away, facilitating the placement of larger corpectomy cages and potentially reducing the risk of complications [10]. Although select evidence suggests a prolonged learning curve and risks in execution, including possible femoral nerve neuropraxia and segmental artery injury, recent studies have found no significantly increased risk for adverse outcomes [14,15,16].

Despite increasing adoption of the prone-transpsoas approach for lumbar interbody fusion, the literature specifically evaluating its use for corpectomy remains limited to technical notes and small case series. Further characterization of perioperative outcomes, technical considerations, and deformity correction potential is therefore needed. The present series aims to contribute additional clinical experience and technical insight regarding PTP-corpectomy for complex thoracolumbar pathology.

## 2. Materials and Methods

### 2.1. Study Design, Setting, and Participants

We retrospectively reviewed seven patients who underwent PTP-corpectomy for various complex spinal pathologies at our institution between 2022 and 2025. Institutional Review Board approval was granted with a waiver of consent. Written consent for media used in figures was obtained. Data were obtained from the electronic medical record and de-identified at collection. This study was conducted in accordance with Health Insurance Portability and Accountability Act (HIPAA) guidelines and reported in accordance with Preferred Reporting of Case Series in Surgery (PROCESS) guidelines [17].

### 2.2. Data Collection

Demographic variables collected included patient age at the time of surgery, sex, and body mass index (BMI). Clinical variables included history of previous spine surgery and pathology, indications for surgery, operative time, estimated blood loss (EBL), length of stay (LOS), postoperative disposition, details of the surgery and perioperative period, as well as radiographic imaging. Operative time was defined by logged anesthesia start and end times. When applicable, X-ray (XR) imaging was used to calculate the sagittal-plane correction, defined as the difference between pre- and postoperative Cobb angles calculated by a single author and verified by two others.

### 2.3. Statistical Analysis

Statistical analysis was performed using R (version 4.1.1, R Foundation for Statistical Computing, Vienna, Austria).

## 3. Results

### 3.1. Case Series

**Figure 1:**
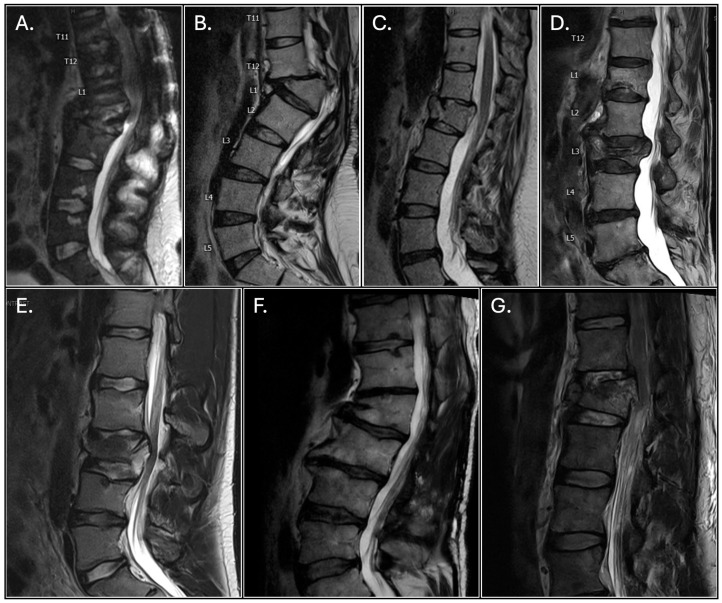
Preoperative MR Imaging. (**A**) Progressive L2 compression fracture with 5 mm retropulsion, focal kyphosis, and canal narrowing. Loosening with bilateral pedicle screw lucency and right cortical breach. Heterotopic ossification spans L1-L3. (**B**) Chronic, severe L1 compression with 13 mm retropulsion, 8 mm distraction, and 5 mm anterior displacement. T12-L2 anterolisthesis, focal kyphosis, and canal stenosis with T2/STIR signal and cord thinning. Arachnoiditis with clumped cauda equina from T11-L2. (**C**) Acute L1 burst fracture with 6 mm retropulsion, 25% height loss, and mild T12-L1 stenosis. Lumbar alignment and conus/nerve roots preserved; no stenosis below L1. (**D**) Chronic L1 compression (45% height loss, residual STIR signal) and acute-on-chronic L3 compression (80% height loss, progressive retropulsion, moderate stenosis). Trace L1-L2 retrolisthesis. (**E**) Comminuted L3 burst fracture with severe retropulsion causing severe canal stenosis. Associated left lamina fracture, right facet dislocation, and retropulsion extending to L2-L4 with foraminal narrowing. L4-L5 disk herniation and facet arthropathy causing additional stenosis. (**F**) Acute comminuted burst fracture of L1 with moderate height loss, and severe canal stenosis due to posterior retropulsion. Focal kyphosis. Epidural hemorrhage extends from T3 to L5. (**G**) L1 burst fracture with moderate height loss and posterior retropulsion causing severe canal stenosis.

**Figure 2:**
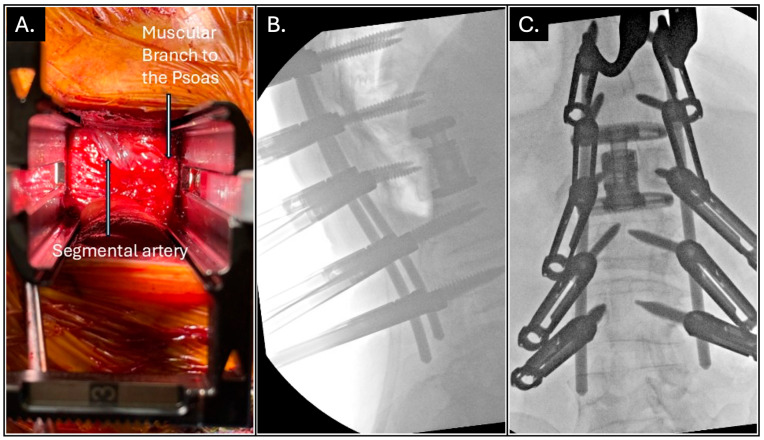
Intraoperative Imaging. Initial lateral exposure and corridor along with retractor setup (**A**). Lateral fluoroscopic XR of the finalized construct in Case F, with posterior instrumentation and corpectomy cage shown (**B**). Posterior-anterior (PA) fluoroscopic XR of the finalized construct of Case F (**C**).

Seven patients (four trauma, three deformity/revision) underwent PTP-corpectomy with posterior fixation between 2022 and 2025. Demographics and preoperative characteristics are summarized in Table 1 and operative and postoperative details in Table 2. Intraoperative navigation was used for all cases. Preoperative imaging is shown in Figure 1 and intraoperative imaging is provided in Figure 2. Intraoperative neuromonitoring was utilized for all seven patients to minimize risk of iatrogenic nerve injury. All patients maintained postoperative stability and improved functional status at latest follow-up (range 41–375 days). One patient required readmission for hematoma/seroma drainage on the posterolateral side overlying the left psoas; however, no neurologic deficits or instrumentation failures were observed.

#### Representative Case (Case D)

A 69-year-old female with osteoporosis presented after a mechanical fall 5 days prior. CT imaging showed multiple vertebral body fractures, most notably an L3 burst fracture. The patient underwent a T12-L5 fusion via a posterior approach with pedicle screw cement augmentation due to vertebral osteoporosis. The PTP approach was used to perform the L3 corpectomy. The procedure was well tolerated, and the patient was transferred to an inpatient rehabilitation center on postoperative day 6 in good condition. At the 3-month follow-up, the patient was noted to be doing clinically well and had improvement in her back and thigh pain.

Detailed case narratives, including mechanism of injury, perioperative course, and rehabilitation details, are provided in Appendix A.

### 3.2. Surgical Technique and Results

#### 3.2.1. Patient Positioning, Navigation, and Posterior Instrumentation

Patients were placed prone on a Jackson surgical table, and coronal-plane bending was used to allow optimal exposure of the lateral side selected for the retroperitoneal or retrodiaphragmatic approach. Normal sterile preparation of the back and flank was performed. Intraoperative CT-guided navigation was used in standard fashion. Standard approaches to posterior instrumentation, either open (*n* = 2, open approach used for revision of prior spinal surgery) or percutaneous (*n* = 5), were used to place pedicle screws and perform osteotomies when needed.

#### 3.2.2. Lateral Approach for Corpectomy

The incision for the lateral approach was planned using intraoperative navigation. The incision was then made and deepened with Bovie electrocautery and blunt dissection. For cases requiring rib resection, the neurovascular bundle was carefully distracted away from the bony anatomy before the rib was resected using rib cutters. When rib resection was not needed, the retroperitoneal space was entered in the standard fashion. For thoracolumbar junction cases involving L1 exposure, the operative corridor was developed either beneath the diaphragm or through careful splitting of diaphragmatic fibers under direct visualization when necessary.

Once the operative space was accessed, the psoas muscle was identified and traversed to expose the vertebral segments of interest. A navigated dilator probe was used to confirm a safe surgical corridor, and a self-retaining retractor was placed to expose the vertebral body for corpectomy. The segmental artery was dissected and coagulated using bipolar cautery. Discectomies were performed at levels adjacent to the corpectomy site.

#### 3.2.3. Corpectomy and Cage Placement

Navigated osteotomes, bone rongeurs, and a high-speed drill were used to resect the vertebral body. Once the corpectomy was completed, an expandable titanium cage was prepared with autograft and allograft, then placed in the defect under fluoroscopic guidance. The cage was expanded, and disk height and alignment were confirmed using fluoroscopy.

#### 3.2.4. Final Fixation, Closure, and Postoperative Management

Once the cage was positioned correctly, a contoured titanium rod was placed with standard technique, and a final check of the instrumentation and alignment was done with fluoroscopy. Meticulous hemostasis and irrigation were performed, along with drain placement when needed. Standard closure of anatomical layers followed for both the posterior and lateral incisions. The patient was then transferred to the PACU or ICU and postoperative care was initiated according to institutional protocols.

### 3.3. Surgical Outcomes

**Figure 3:**
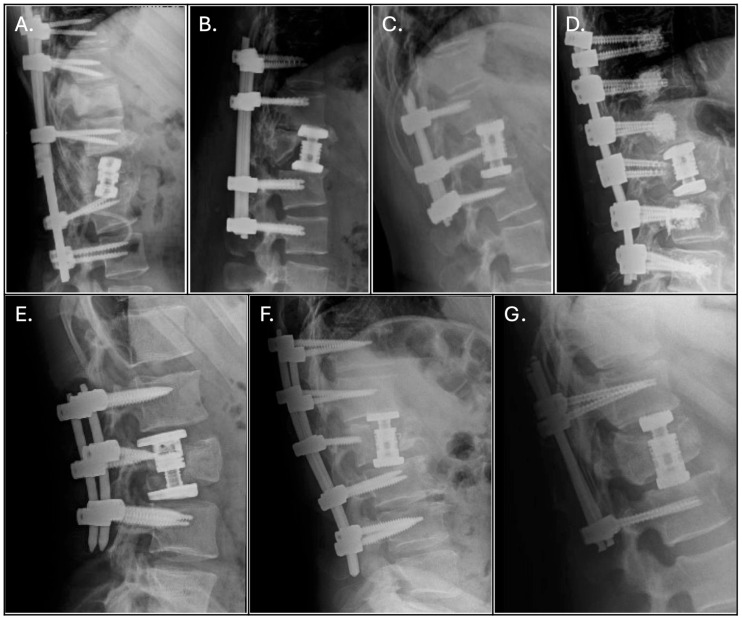
Postoperative XR Imaging. Each postoperative X-ray corresponds with the preoperative MR imaging in the same lettered panel in Figure 1. (**A**) Posterior fusion from T10 to L4 with L2 corpectomy and cage spanning L1-L3. T12 compression fracture persists with moderate height loss and retropulsion (3 months post-op). (**B**) Partial L1 corpectomy with anterior strut graft and posterior fusion from T11 to L3. Residual L1 fracture fragment remains. Other vertebral bodies are aligned with preserved height and disk spaces (post-op day 4). (**C**) L1 corpectomy with posterior fusion from T12 to L2. Vertebral alignment and heights are stable compared to prior imaging (3 months post-op). (**D**) Partial L3 corpectomy and posterior fusion from T12 to L5. Cement augmentation is present at screw tips. L3 compression fracture and L1 endplate deformity are stable. Disk space loss persists at L5-S1 (3 months post-op). (**E**) Partial L3 corpectomy and posterior fusion from L2 to L4. L3 burst fracture remains moderately compressed with mild retropulsion and anterior cortical displacement (2 months post-op). (**F**) Posterior fusion from T11 to L3 with L1 partial corpectomy and cage spanning from endplates of T12 to L2 (6 weeks post-op). (**G**) Posterior fusion from T12 to L2 with L1 corpectomy.

The PTP-corpectomy procedure was well tolerated by all seven patients in our study. Six of the seven patients were noted to be doing well and participating in physical therapy at 3-month follow-up office visits, while one patient (Case B) was lost to follow-up with our surgical team after 5 weeks of recovery. Immediate postoperative imaging for this patient, shown in Figure 3, revealed stable and intact instrumentation with placement as intended. For the six patients with later follow-up imaging available, the instrumentation continued to appear stable and intact (Figure 3). One patient required surgical drainage of a seroma/hematoma over the surgical site, but otherwise, there were no notable postoperative complications for any of the seven patients. Overall, the procedure was well tolerated and improved clinical status of each patient was observed.

Surgical and postoperative details are summarized in Table 2. The average operative time was 460.6 ± 147.1 min, and the estimated blood loss (EBL) was 892.9 ± 898.3 mL. The average length of stay (LOS) postoperatively was 6.7 ± 3.0 days. One case required revision of a preexisting construct and complex wound closure with plastic surgery, which had significantly increased operative time and blood loss (767 min, 2700 mL). Excluding this complicated case, the average time was 409 ± 63.7 min, and EBL was 591.7 ± 454.3 mL. All seven patients maintained clinical stability, demonstrating improvements in pain and functional status at latest follow-up. Follow-up time ranged from 41 to 375 days. Six of seven patients had clinical follow-up through at least postoperative week 6, which was considered sufficient to assess early construct stability, perioperative complications, and initial functional recovery. One patient was lost to follow-up after 41 days but had reported reduced pain at discharge.

## 4. Discussion

### 4.1. Advantages of the PTP Approach for Corpectomy

Our experience suggests that PTP-corpectomy is a feasible and efficient technique for managing complex spinal pathologies requiring vertebral body resection and reconstruction. PTP-corpectomy also proved to be an effective approach for the three cases in which previous surgical intervention had failed (Table 1). Multiple authors have highlighted the versatility of the PTP approach for various spinal pathologies including trauma, advanced degenerative disease, and complex revision scenarios [10,14]. The presented series included both burst fractures and compression fractures, with preoperative imaging and case descriptions shown in Figure 1 and Table 1, respectively. As discussed by Gandhi et al., the PTP approach provides the ability to address both anterior and posterior aspects of the vertebral column using only the prone position, which is especially beneficial in cases where considerable vertebral body compromise necessitates corpectomy [13].

**Figure 4:**
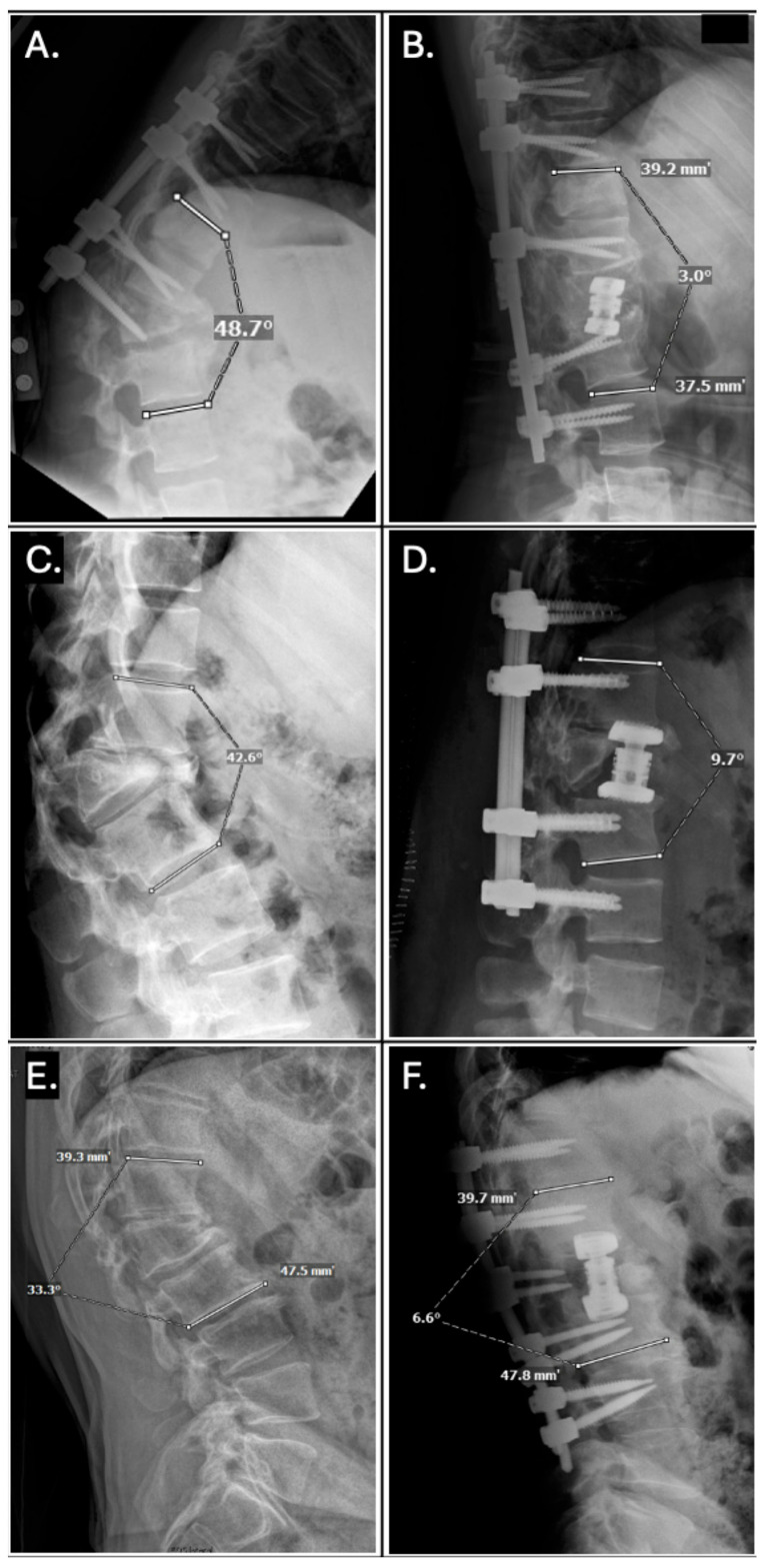
Sagittal-Plane Correction. Case A pre- (**A**) and postoperative (**B**) XR imaging, showing a final sagittal-plane alignment correction of 45.7 degrees. Case B pre- (**C**) and postoperative (**D**) XR imaging 32.9 degrees of sagittal-plane alignment correction. Case F pre- (**E**) and postoperative (**F**) XR imaging 26.7 degrees of sagittal-plane alignment correction.

Several advantages of the PTP approach have been reported. First, avoiding repositioning from lateral decubitus to prone often reduces operative time, lowering anesthesia needs and potentially infection risk [14,18]. Further, the prone position tends to facilitate sagittal-plane correction and indirect decompression, as the prone positioning naturally places the patient into a more lordotic alignment compared to the lateral decubitus position [10]. Walker et al. compared single-position PTP fusion to the standard dual-position LLIF and found that the single-position prone approach provided a significantly larger improvement in the segmental lordotic angle (5.1° vs. 2.5°, *p* = 0.02) [19]. Our experience supports the use of the PTP approach for maximizing the correction of sagittal-plane alignment. In the three cases necessitating alignment correction in our series, we obtained an average improvement in Cobb angle of 35.1 ± 9.7 degrees (Figure 4; Table 2). This substantial correction highlights the utility of PTP-corpectomy in correcting sagittal-plane alignment. Third, the prone positioning in the PTP approach causes a beneficial shift in anatomical structures. A study comparing anatomical positions used in LLIF found that the lateral decubitus position shifts the psoas muscle anteriorly, bringing the femoral nerve anteriorly along with it [20]. Comparatively, the prone position shifts the psoas muscle and femoral nerve posteriorly. The authors therefore theorized that the prone position may be advantageous for avoiding femoral nerve injury [20]. With the posterior shift in the psoas muscle and anterior shift in soft tissue structures due to gravity in the prone positioning, our experience has shown that the prone positioning affords a more spacious lateral corridor for instrumentation and corpectomy (Figure 2). This wide lateral corridor enables the placement of larger cages, which other studies have found to improve load distribution, overall strength of the construct, and resistance to migration [21].

Several studies have also compared the adverse event profile of the PTP approach to traditional approaches such as the lateral decubitus positioning with and without a position change to prone. In their 2021 multicenter cohort study, Smith et al. concluded that the PTP approach is both feasible and efficient as it allows for single-position surgery with maximized circumferential access and correction of alignment, as well as perioperative outcomes comparable to the lateral decubitus position [22]. These findings were validated by several studies, including both a systematic review with pooled analysis [16], an additional multicenter study [15], and a survey of early adopters of the approach [23], which found the PTP approach to be associated with significant improvement in clinical outcomes and a safety profile comparable to widely adopted techniques.

Prior studies evaluating lumbar and thoracolumbar corpectomies have reported a range of operative times, estimated blood loss (EBL), and length of stay (LOS) across techniques. In a systematic review of 702 lumbar corpectomy patients, Wipplinger et al. reported that anterolateral approaches had longer operative times and greater EBL compared to posterolateral approaches (317 ± 178 min and 1393 ± 1341 mL versus 258 ± 93 min and 982 ± 567 mL, respectively) [24]. Of note, anterolateral approaches often required repositioning for posterior instrumentation, adding to both operative time and LOS, while complication and revision rates were higher in the posterolateral cohort [24]. In a similar study, however, Chiu et al. reported no significant differences in LOS in their analysis of 1327 anterior- and posterior-approach thoracic corpectomies [25]. Prone-transpsoas techniques have shown some promise in improving these endpoints as well, with groups reporting shorter (albeit non-significant) operative times and LOS for prone-transpsoas LLIF compared to lateral decubitus LLIF, while permitting reduced repositioning [22,23,26]. It is worth noting that the cases in our series had an average blood loss of 892.9 mL and operative time of 460.6 min, which highlights the complexity of the PTP-corpectomy procedure. However, our experience suggests that these metrics may improve as surgeons progress through the learning curve. Our findings add to the existing literature, suggesting that PTP-corpectomy offers operative efficiency without increasing morbidity, in part by eliminating the need for intraoperative repositioning.

### 4.2. Potential Limitations and Learning Curves

Despite a favorable complication profile, there are potential risks associated with the PTP approach, both for fusion and corpectomy. The PTP approach’s unique patient positioning, which enables simultaneous lateral and posterior access, introduces a learning curve that surgeons adopting the technique should be prepared to navigate [14,16]. The segmental artery is easily damaged and must be identified early in the surgery and appropriately divided. Surgeons must also stay alert to avoid excessive psoas or lumbar plexus traction, which can lead to nerve-related complications such as femoral nerve neuropraxia [14,27]. Moreover, operating in the prone position can be technically challenging in obese or anatomically complex patients, and access can be limited, particularly at the L5-S1 level due to the iliac crest [10]. Although the PTP approach has been shown to have no significant increase in adverse outcomes, we believe continued scrutiny of patient selection, outcomes, and complications is warranted.

### 4.3. Indications and Contraindications

Given the unique advantages and challenges associated with the PTP approach for lumbar corpectomy, patients must be selected after careful consideration of their broader clinical profiles. Indications for PTP-corpectomy include the need for circumferential access to the vertebral column for decompression, alignment correction, and fusion in patients refractory to conservative treatment [1,3,10]. Contraindications often include prior retroperitoneal surgery with significant scarring, active infection, severe osteoporosis, and anatomical barriers such as a high iliac crest [10,28,29]. However, in our experience, the PTP-corpectomy procedure was successfully completed on a patient with an active wound infection from a prior spine surgery (Case A) and employed cement augmentation while performing PTP-corpectomy on a patient with osteoporosis (Case D) (detailed narratives available in Appendix A). Moreover, two studies found that the PTP approach can safely provide greater access to the L4-5 level compared to the lateral decubitus position [29,30]. This suggests potential feasibility of PTP-corpectomy at this level; however, we did not operate at this level in our case series. Relative contraindications include morbid obesity, severe vascular anomalies, or inability to tolerate prone positioning [10,23,28]. Preoperative imaging should confirm the feasibility of creating a sufficient lateral corridor and the absence of vascular or visceral structures at risk. The patients within our case series required circumferential access to the vertebral column to resect the deformed vertebral body, restore stability, and, when applicable, provide significant correction of alignment.

### 4.4. Technology and Future Directions

All seven PTP-corpectomy cases included in our study utilized intraoperative navigation during the procedure. The use of intraoperative navigation in the PTP approach for lumbar interbody fusion and traditional approaches for corpectomy has previously been described by several studies, but is sparsely described for PTP-corpectomy [31,32,33,34]. Due to the unique anatomical positioning, limited direct visualization, and challenging ergonomics, the real-time guidance provided by intraoperative navigation is especially valuable for the PTP approach [28]. Moreover, navigation can decrease reliance on traditional fluoroscopy, minimizing radiation exposure to the patient and surgical team [34]. In our case series, navigation was crucial for safe instrumentation placement, planning the incision, and directing the osteotomy cuts for the corpectomy.

Overall, our findings support the use of the PTP approach for single-position corpectomy. The presented cases demonstrate that PTP-corpectomy can effectively manage complex spinal pathology and facilitate substantial corrections in alignment while avoiding the drawbacks associated with traditional approaches. The prone-transpsoas approach represents an innovative advancement in single-position spine surgery with promising applications for complex thoracolumbar reconstruction and deformity correction.

### 4.5. Limitations

This series adds to the limited case volume describing this technique in the current literature. However, this study is also limited by the highly heterogeneous and small cohort.

Additional studies are necessary to further validate these findings, delineate optimal patient selection criteria, and quantify the comparative benefits and limitations of the PTP approach relative to traditional surgical strategies.

## 5. Conclusions

Our series provides encouraging support for the PTP-corpectomy procedure as an effective surgical technique for managing complex spinal pathology requiring lateral corpectomy and posterior stabilization.

## Figures and Tables

**Table 1:** Patient Demographics and Preoperative Status.

Case	Age	Sex	BMI	Pertinent PMH	Primary Diagnosis	Preoperative Neurologic/Functional Status
A	23	F	23.50	History of T10 burst fracture treated at outside hospital; chronic bilateral lower extremity paralysis; wheelchair bound	L2 compression fracture with worsening thoracolumbar kyphoscoliosis; wound dehiscence, hardware exposure	Wheelchair use hindered by severe kyphoscoliosis
B	41	F	26.06	History of traumatic L1 compression fracture and L1 laminectomy at outside hospital; chronic bilateral lower extremity paralysis; neurogenic bowel/bladder on chronic foley; chronic kyphoscoliosis; wheelchair bound	Chronic L1 compression fracture with retropulsion causing severe stenosis; severe kyphoscoliosis	Wheelchair use hindered by severe kyphoscoliosis and back pain
C	45	F	25.51	No significant medical or surgical history	L1 burst fracture	Full neurologic and functional status
D	69	F	15.12	Osteoporosis	Multiple vertebral body fractures including L3 burst fracture causing severe stenosis	Full neurologic and functional status
E	17	M	25.11	No significant medical or surgical history	L3 Chance fracture and three-column injury; urinary retention and severe spinal canal stenosis seen on imaging	Full functional status; concern for cauda equina syndrome due to high post-void urinary retention
F	59	M	25.49	History of L1 fracture with progressing lumbar kyphoscoliosis and stenosis; multiple unsuccessful previous lumbar decompressions	L1 vertebral body fracture with progressive lumbar kyphoscoliosis, back pain and bilateral lower extremity pain	Symptoms of neurogenic claudication; lower back and extremity pain
G	46	M	32.78	No significant medical or surgical history	L1 burst fracture	Full neurologic and functional status

BMI, body mass index; PMH, past medical history.

**Table 2:** Surgical and Postoperative Details.

Case	Surgical Levels	Op Time (min)	EBL (mL)	LOS (Days)	Disposition	Sagittal Plane Correction	Postoperative Neurologic/Functional Status	Postoperative Complications	Readmit (30 Days)	Reop	Follow-Up Time (Days)
A	T10-L4; L2 corpectomy	767	2700	9	Home with home health services	45.7	Corrected and stabilized thoracolumbar alignment; reduction in back pain	None	No	No	375
B	T11-L3; L1 corpectomy	492	1000	7	Inpatient rehab	32.9	Corrected and stabilized thoracolumbar alignment; reduction in back pain	UTI, pyelonephritis	Yes	No	41
C	T12-L2; L1 corpectomy	337	200	5	Home	N/A	No changes; maintained full neurologic and functional status	Left flank numbness	No	No	105
D	T12-L5; L3 corpectomy	463	500	5	Inpatient rehab	N/A	Reduction in stenosis symptoms; maintained full neurologic and functional status	Left anterior thigh burning pain	No	No	183
E	L2-L4; L3 corpectomy	382	1300	3	Home	N/A	Resolved urinary retention concerning for cauda equina syndrome; maintained full functional status	Intermittent low back tightness; improving with time	No	No	92
F	T11-L3; L1 corpectomy	436	300	6	Home with home health services	26.7	Neurogenic claudication symptoms improved (right-sided back pain resolved); maintained full functional status	Left groin and flank pain due to hematoma/seroma	Yes	Yes	47
G	T12-L2; L1 corpectomy	347	250	12	Inpatient rehab	N/A	No changes; maintained full neurologic and functional status	None	No	No	192

Op Time, operative time; EBL, estimated blood loss; Readmit, readmission; Reop, reoperation; N/A, not applicable. Operative time was calculated as the difference between anesthesia start and stop times.

## Data Availability

The original contributions presented in this study are included in the article. Further inquiries can be directed to the corresponding author.

## Abbreviations

The following abbreviations are used in this manuscript:

PTP	Prone-transpsoas
PTP-corpectomy	Prone-transpsoas corpectomy
LLIF	Lateral lumbar interbody fusion
IRB	Institutional Review Board
HIPAA	Health Insurance Portability and Accountability Act
PROCESS	Preferred Reporting Of Case Series in Surgery
BMI	Body mass index
EBL	Estimated blood loss
LOS	Length of stay
XR	X-ray
CT	Computed tomography
MRI	Magnetic resonance imaging
PACU	Post-anesthesia care unit
ICU	Intensive care unit

## Appendix A

### Case Narratives

Case A

A 23-year-old female with a history of T10-L2 posterior spinal fusion with bilateral decompression from T11-L1 for a T12 burst fracture 3 months prior presented with 1 week of wound dehiscence and hardware exposure. Neurological exam revealed complete (0/5) chronic bilateral lower extremity paralysis and sensory deficit at T10. Imaging showed worsening thoracolumbar kyphoscoliosis with an L2 compression fracture. She underwent surgical wound revision, hardware removal, extended posterior decompression to L4, and an L2 corpectomy with placement of an expandable titanium cage via the PTP approach. Postoperatively, she was discharged on day 6. At 3-month follow-up, she was ambulatory using a wheelchair and a back brace and engaging in physical therapy; imaging showed stable cage and hardware alignment. Clinical status remained stable at 6-month and 1-year follow-ups.

Case B

A 41-year-old female with chronic severe lower back and bilateral gluteal pain radiating down both legs presented after acute worsening of her pain that prevented wheelchair use. Eight years prior, she sustained a traumatic L1 compression fracture from a three-story fall, resulting in spinal cord injury and thoracolumbar kyphoscoliosis. She underwent an L1 laminectomy at an outside hospital but was ultimately left wheelchair-bound with chronic kyphoscoliosis. Neurological exam revealed chronic bilateral motor and sensory deficits in her lower extremities. Imaging showed chronic L1 compression fracture with retropulsion causing severe stenosis, and T11-12 STIR signal suggestive of myelomalacia or arachnoiditis. With the goal of correcting the kyphoscoliosis and enabling wheelchair use, she underwent L1 corpectomy via the PTP approach, with simultaneous posterior T11-L3 fusion. She was discharged in stable condition to inpatient rehabilitation 1-week after surgery, noting an improvement in her back pain. During postoperative week 2, she returned to the ED and was ultimately readmitted due to cystitis and pyelonephritis secondary to chronic intermittent self-catheterization. This was managed with IV antibiotics, and she was discharged to home in stable condition by postoperative week 5. She was subsequently lost to further follow-up.

Case C

A 44-year-old female presented after a mechanical fall. Imaging revealed an L1 burst fracture. The patient underwent an L1 corpectomy performed via the PTP approach, with simultaneous posterior T12-L2 fusion. The procedure was well tolerated, and the patient was discharged to home on postoperative day 4. At 6-week follow-up, the patient was noted to be ambulating daily and denied pain, but did report mild numbness in the left flank and lower back away from the incision site. Upright X-rays showed intact hardware. The patient began physical therapy shortly thereafter.

Case D

A 69-year-old female with osteoporosis presented after a mechanical fall 5 days prior. CT imaging showed multiple vertebral body fractures, most notably an L3 burst fracture. The patient underwent a T12-L5 fusion via a posterior approach with pedicle screw cement augmentation due to vertebral osteoporosis. The PTP approach was used to perform the L3 corpectomy. The procedure was well tolerated, and the patient was transferred to an inpatient rehabilitation center on postoperative day 6 in good condition. At the 3-month follow-up, the patient was noted to be doing clinically well and had improvement in her back and thigh pain.

Case E

A 17-year-old male presented with an L3 Chance fracture and three-column injury after a motor vehicle accident. Clinical imaging and examination suggested cauda equina syndrome. Due to the unstable fracture, the patient underwent urgent decompression and fixation with an L2-L4 fusion and L3 corpectomy using the PTP approach. He was discharged to home on postoperative day 2. At 3-month follow-up, he was clinically stable with mild, but improving, left thigh numbness.

Case F

A 59-year-old male with a remote history of an L1 vertebral body fracture, progressive lumbar kyphoscoliosis, and stenosis presented with recurrent neurogenic claudication after two lumbar decompressions failed to provide lasting relief 6 months and 14 months prior. CT and XR imaging demonstrated an L1 compression deformity and 41 degrees of kyphosis centered around L1. The patient underwent a T11-L3 posterior spinal fusion with L1 corpectomy via the PTP approach. The procedure was well tolerated, and the patient was discharged home in stable condition on postoperative day 5. The patient was readmitted on postoperative day 14 for back pain and subsequently underwent surgical drainage of a hematoma/seroma over the left psoas. At 6 weeks follow-up, the patient was noted to be doing clinically well.

Case G

A 46-year-old male presented with coccygeal pain following a fall off a 6-foot ladder. CT imaging revealed an L1 burst fracture. Patient underwent a T12-L2 posterior spinal fusion with L1 corpectomy via the PTP approach. The procedure was well tolerated, and the patient was discharged to inpatient rehabilitation in stable condition on postoperative day 7. At the 6-week follow-up, the patient had been discharged from inpatient rehabilitation and reported significant improvements in ambulation. The patient also reported issues with urinary incontinence and sexual dysfunction, which were attributed to the spinal cord injury from the initial fall. At 6-month follow-up, the patient noted continued improvement in mobility and ability to perform activities of daily living. He reported occasional mild-to-moderate discomfort with repetitive tasks and spontaneous movements, as well as continuing urinary incontinence and sexual dysfunction.
